# Global Stability of HIV Infection of CD4^+^ T Cells and Macrophages with CTL Immune Response and Distributed Delays

**DOI:** 10.1155/2013/653204

**Published:** 2013-12-02

**Authors:** A. M. Elaiw, R. M. Abukwaik, E. O. Alzahrani

**Affiliations:** ^1^Department of Mathematics, Faculty of Science, King Abdulaziz University, P.O. Box 80203, Jeddah 21589, Saudi Arabia; ^2^Department of Mathematics, Faculty of Science, Al-Azhar University, Assiut 71511, Egypt

## Abstract

We study the global stability of a human immunodeficiency virus (HIV) infection model with Cytotoxic T Lymphocytes (CTL) immune response. The model describes the interaction of the HIV with two classes of target cells, CD4^+^ T cells and macrophages. Two types of distributed time delays are incorporated into the model to describe the time needed for infection of target cell and virus replication. Using the method of Lyapunov functional, we have established that the global stability of the model is determined by two threshold numbers, the basic reproduction number *R*_0_ and the immune response reproduction number *R*_0_^∗^. We have proven that, if *R*_0_ ≤ 1, then the uninfected steady state is globally asymptotically stable (GAS), if *R*_0_* ≤ 1 < *R*_0_, then the infected steady state without CTL immune response is GAS, and, if *R*_0_* > 1, then the infected steady state with CTL immune response is GAS.

## 1. Introduction

One of the most diseases that have attracted the attention of many mathematicians is the acquired immunodeficiency syndrome (AIDS) caused by human immunodeficiency virus (HIV). HIV infects the CD4^+^ T cell which plays the central role in the immune system. Mathematical modeling and model analysis of HIV dynamics are important to discover the dynamical behaviors of the viral infection process and estimating key parameter values which leads to development of efficient antiviral drug therapies. Several mathematical models have been proposed to describe the HIV dynamics with CD4^+^ T cells [1–15]. In these papers, the Cytotoxic T Lymphocytes (CTL) immune response was not taken into account. The role of CTL is universal and necessary to eliminate or control the disease during viral infections. In particular, as a part of innate response, CTL plays a particularly important rate in antiviral defense by attacking infected cells. The basic HIV infection model which takes into consideration the CTL immune response has been proposed in [16] as

(1) $$\begin{array}{c} \dot{x}\left(t\right)=\lambda -dx\left(t\right)-\beta x\left(t\right)v\left(t\right), \end{array}$$

(2) $$\begin{array}{c} \dot{y}\left(t\right)=\beta x\left(t\right)v\left(t\right)-ay\left(t\right)-py\left(t\right)z\left(t\right), \end{array}$$

(3) $$\begin{array}{c} \dot{v}\left(t\right)=ky\left(t\right)-rv\left(t\right), \end{array}$$

(4) $$\begin{array}{c} \dot{z}\left(t\right)=cy\left(t\right)z\left(t\right)-bz\left(t\right). \end{array}$$

The state variables describe the plasma concentrations of  *x*(*t*), the uninfected CD4^+^ T cells;  *y*(*t*), the infected CD4^+^ T cells;  *v*(*t*), the free virus particles; and  *z*(*t*), the CTL cells at time  *t*. Here, (1) describes the population dynamics of the uninfected CD4^+^ T cells, where  *λ*  represents the rate of new uninfected cells that are generated from sources within the body,  *d*  is the death rate constant, and  *β*  is the infection rate constant at which a target cell becomes infected via contacting with virus. Equation (2) describes the population dynamics of the infected CD4^+^ T cells and shows that they die with rate constant  *a*  and are killed by the CTL immune response with rate constant  *p*. Equation (3) describes the population dynamics of the free virus particles and shows that they are produced by the infected cells with rate constant  *k*  and removed from the body with rate constant  *r*. Equation (4) describes the population dynamics of the CTL cells which are produced with rate constant  *c*  and die with rate constant  *b*. Model (1)–(4) is based on the assumption that, once the virus contacts a target cell, the cell begins producing new virus particles. However, as pointed by Li and Shu [17], the period between the time for HIV to enter the target cell and the time for new virions to be produced from the infected cell needs the following stages: (i) the period between the viral entry of a target cell and integration of viral DNA into the host genome, (ii) the period from the integration of viral DNA to the transcription of viral RNA and translation of viral proteins such as reverse transcriptase, integrase, and protease, and (iii) the period between the transcription of viral RNA and the release and maturation of virus [17]. More realistic models incorporate the delay between the time of viral entry into the target cell and the time of the production of new virus particles, modeled with discrete time delay or distributed time delay (see, e.g., [3–7]). In [3–7], the HIV infection models did not take into account the impact of the immune response. The time delay has been incorporated into the HIV infection models with CTL immune response in [18–22]. It was assumed that the HIV attacks one class of target cells, CD4^+^ T cells. In 1997, Perelson et al. [23], observed that the HIV attacks two classes of target cells, CD4^+^ T cells and macrophages. HIV infection models with two classes of target cells, CD4^+^ T cells and macrophages, have been proposed in [1, 2, 8, 9, 11]; however, the effect of CTL immune response was neglected. In [24, 25], HIV infection models with two classes of target cells and with CTL immune response have been proposed. In [24], one type of discrete delay (stages (i) and (ii)) has been incorporated into the model. However, it is more realistic to consider the second type of delays between viral RNA transcription and viral release and maturation.

The purpose of the present paper is to propose an HIV infection model with two classes of target cells and two types of distributed delays taking into consideration the CTL immune response. The global stability of the steady state of the model are established using Lyapunov functional. We have proven that the global dynamics of this model is determined by the basic reproduction number  *R*_0_  and the immune response reproduction number  *R*_0_*. We have shown that, if  *R*_0_ ≤ 1, then the uninfected steady state is globally asymptotically stable (GAS), if  *R*_0_* ≤ 1 < *R*_0_, then the infected steady state without CTL immune response is GAS, and, if  *R*_0_* > 1, then the infected steady state with CTL immune response is GAS.

### 1.1. The Model

In this section, we propose a mathematical model of HIV infection which describes two cocirculation populations of target cells, potentially representing CD4^+^ T cells and macrophages, taking into account the CTL immune response and multiple distributed intracellular delays

(5) $$\begin{array}{c} {\dot{x}}_{i}\left(t\right)=\lambda_{i}-d_{i}x_{i}\left(t\right)-\beta_{i}x_{i}\left(t\right)v\left(t\right),\;i=1,2, \end{array}$$

(6) y˙i(t)=βi∫0hifi(τ)e−miτxi(t−τ)v(t−τ)dτ −ayi(t)−pyi(t)z(t), i=1,2,

(7) $$\begin{array}{c} \dot{v}\left(t\right)=k\int_{0}^{h_{3}}g\left(\tau \right)e^{-n\tau }\sum_{i=1}^{2}y_{i}\left(t-\tau \right)d\tau -rv\left(t\right), \end{array}$$

(8) $$\begin{array}{c} \dot{z}\left(t\right)=c\sum_{i=1}^{2}y_{i}\left(t\right)z\left(t\right)-bz\left(t\right). \end{array}$$

Here  *i* = 1,2  corresponds to the CD4^+^ T cells and macrophages, respectively. All the variables and parameters of the model have the same meanings as given in (1)–(4). To take into account the delay between viral infection of an uninfected target cell and the production of an actively infected target cell, we let  *τ*  be the random variable that describes the time between viral entry and the transcription of viral RNA (stages (i) and (ii)) with a probability distribution  *f*_*i*_(*τ*)  over the interval  [0, *h*_*i*_], and  *h*_*i*_  is limit superior to this delay. The factor  *e* ^−*m*_*i*_*τ*^  accounts for the loss of target cells during this delay period, where  *m*_*i*_  is constant. On the other hand, to consider the delay between viral RNA transcription and viral release and maturation, we let  *τ*  be the random variable; that is, the time between these two events with a probability distribution  *g*(*τ*)  over the interval  [0, *h*_3_], and  *h*_3_  is limit superior to this delay [17]. The factor  *e* ^−*nτ*^  accounts for the loss of infected cells during this delay period, where  *n*  is constant.

The probability distribution functions  *f*_*i*_(*τ*),  *i* = 1,2, and  *g*(*τ*)  are assumed to satisfy  *f*_*i*_(*τ*) > 0, and  *g*(*τ*) > 0, and

(9) $$\begin{array}{c} \int_{0}^{h_{i}}f_{i}\left(\tau \right)d\tau =1,\;\int_{0}^{h_{i}}f_{i}\left(r\right)e^{sr}dr<\infty ,\;i=1,2,\\ \int_{0}^{h_{3}}g\left(\tau \right)d\tau =1,\;\;\int_{0}^{h_{3}}g\left(r\right)e^{sr}dr<\infty , \end{array}$$

where  *s*  is a positive constant. Let

(10) $$\begin{array}{c} F_{i}=\int_{0}^{h_{i}}f_{i}\left(\tau \right)e^{-m_{i}\tau }d\tau ,\;i=1,2,\\ G=\int_{0}^{h_{3}}g\left(\tau \right)e^{-n\tau }d\tau . \end{array}$$

Then

(11) $$\begin{array}{c} 0<F_{i}\leq 1,\;0<G\leq 1,\;i=1,2. \end{array}$$

The initial conditions for system (5)–(8) take the form

(12) $$\begin{array}{c} x_{1}\left(\theta \right)=\varphi_{1}\left(\theta \right),\;\;y_{1}\left(\theta \right)=\varphi_{2}\left(\theta \right),\\ x_{2}\left(\theta \right)=\varphi_{3}\left(\theta \right),\;\;y_{2}\left(\theta \right)=\varphi_{4}\left(\theta \right),\\ v\left(\theta \right)=\varphi_{5}\left(\theta \right),\;\;z\left(\theta \right)=\varphi_{6}\left(\theta \right),\\ \varphi_{j}\left(\theta \right)\geq 0,\;\theta \in \left[-h,0\right),\;\;j=1,\ldots ,6,\\ \varphi_{j}\left(0\right)>0,\;j=1,\ldots ,6, \end{array}$$

where  *h* = max⁡{*h*_1_, *h*_2_, *h*_3_}  and  (*φ*_1_(*θ*), *φ*_2_(*θ*),…, *φ*_6_(*θ*)) ∈ *C*([−*h*, 0], *R*_+_^6^), where  *C*  is the Banach space of continuous functions mapping the interval  [−*h*, 0]  into  *R*_+_^6^. By the fundamental theory of functional differential equations [26], system (5)–(8) have a unique solution satisfying the initial conditions (12).

### 1.2. Nonnegativity and Boundedness of Solutions

In the following, we establish the nonnegativity and boundedness of solutions of (5)–(8) with initial conditions (12).

**Proposition**

Let  (*x*_1_(*t*), *y*_1_(*t*), *x*_2_(*t*), *y*_2_(*t*), *v*(*t*), *z*(*t*))  be any solution of (5)–(8) satisfying the initial conditions (12); then  *x*_1_(*t*), *y*_1_(*t*), *x*_2_(*t*), *y*_2_(*t*), *v*(*t*), and  *z*(*t*)  are all non-negative for  *t* ≥ 0  and ultimately bounded.

**Proof**

First, we prove that  *x*_*i*_(*t*) > 0,  *i* = 1,2, for all  *t* ≥ 0.  Assume that  *x*_*i*_(*t*)  loses its nonnegativity on some local existence interval  [0, *ρ*]  for some constant  *ρ*  and let  *t*_i_* ∈ [0, *ρ*]  be such that  *x*_*i*_(*t*_i_*) = 0.  From (5) we have$\;{\dot{x}}_{i}(t_{i}^{*})=\lambda_{i}>0.\;$Hence  *x*_*i*_(*t*) > 0  for some  *t* ∈ (*t*_i_*, *t*_i_* + *ϵ*), where  *ϵ* > 0  is sufficiently small. This leads to a contradiction and hence  *x*_*i*_(*t*) > 0, for all  *t* ≥ 0.  Further, from (6) and (7) we have

(13) yi(t)=yi(0)e−∫0t[a+pz(ξ)]dξ +βi∫0te−∫ηt[a+pz(ξ)]dξ      ×∫0hifi(τ)e−miτxi(η−τ)v(η−τ)dτ dη,                       i=1,2,v(t)=v(0)e−rt +k∫0te−r(t−η)∫0h3g(τ)e−nτ∑i=12yi(η−τ)dτ dη,

confirming that  *y*_*i*_(*t*) ≥ 0,  *i* = 1,2, and  *v*(*t*) ≥ 0  for all  *t* ∈ [0, *h*]. By a recursive argument, we obtain  *y*_*i*_(*t*) ≥ 0,  *i* = 1,2, and  *v*(*t*) ≥ 0  for all  *t* ≥ 0.  Now from (8) we have

(14) $$\begin{array}{c} z\left(t\right)=z\left(0\right)e^{-bt+c\sum_{i=1}^{2}\int_{0}^{t}y_{i}(\eta )d\eta }. \end{array}$$

Then  *z*(*t*) ≥ 0, for all  *t* ≥ 0.

Next we show the boundedness of the solutions of system (5)–(8). From (5) we have$\;{\dot{x}}_{i}(t)\leq \lambda_{i}-d_{i}x_{i}(t)$,  *i* = 1,2. This implies  lim⁡sup⁡_*t*→*∞*_ *x*_*i*_(*t*) ≤ *λ*_*i*_/*d*_*i*_,  *i* = 1,2.

Let  *X*(*t*) = ∑_*i*=1_^2^[*F*_*i*_ *x*_*i*_(*t*) + *y*_*i*_(*t* + *τ*)] + (*p*/*c*)*z*(*t* + *τ*). Then

(15) X˙(t)=∑i=12[Fiλi−Fidixi(t)−Fiβixi(t)v(t)+Fiβixi(t)v(t)   −ayi(t+τ)−pyi(t+τ)z(t+τ)] +p∑i=12yi(t+τ)z(t+τ)−pcbz(t+τ)=∑i=12[Fiλi−Fidixi(t)−ayi(t+τ)]−pcbz(t+τ)≤∑i=12Fiλi−σ(∑i=12[Fixi(t)+yi(t+τ)]+pcz(t+τ))=∑i=12Fiλi−σX(t),

where  *σ* = min⁡{*d*_1_, *d*_2_, *a*, *b*}. Hence  lim⁡sup⁡_*t*→*∞*_ *X*(*t*) ≤ *L*, where  *L* = ∑_*i*=1_^2^ *L*_*i*_ = ∑_*i*=1_^2^(*F*_*i*_ *λ*_*i*_/*σ*). Since  *x*_*i*_(*t*) > 0,  *y*_*i*_(*t*) ≥ 0  and  *z*(*t*) ≥ 0 then  lim⁡sup⁡_*t*→*∞*_∑_*i*=1_^2^ *y*_*i*_(*t*) ≤ *L*,  lim⁡sup⁡_*t*→*∞*_ *y*_*i*_(*t*) ≤ *L*,  *i* = 1,2  and  lim⁡sup⁡_*t*→*∞*_ *z*(*t*) ≤ *cL*/*p*. On the other hand,

(16) v˙(t)≤k∫0h3g(τ)e−nτ(L)dτ−rv=kGL−rv.

Then  lim⁡sup⁡_*t*→*∞*_ *v*(*t*) ≤ *kGL*/*r*. Therefore,  *x*_1_(*t*), *y*_1_(*t*), *x*_2_(*t*), *y*_2_(*t*), *v*(*t*), and  *z*(*t*)  are ultimately bounded.

### 1.3. Steady States

First we define the basic reproduction number  *R*_0_  and immune response reproduction number  *R*_0_*  of system (5)–(8) as

(17) $$\begin{array}{c} R_{0}=\sum_{i=1}^{2}R_{0i}=\sum_{i=1}^{2}\frac{kGF_{i}\beta_{i}\lambda_{i}}{ard_{i}},\\ R_{0}^{*}=\sum_{i=1}^{2}\frac{F_{i}\beta_{i}\lambda_{i}kGc}{a\left(d_{i}rc+\beta_{i}kGb\right)}. \end{array}$$

We can rewrite  *R*_0_*  as

(18) $$\begin{array}{c} R_{0}^{*}=\sum_{i=1}^{2}R_{0i}^{*}=\sum_{i=1}^{2}\frac{I_{i}R_{0i}}{I_{i}+R_{0i}/F_{i}},\;I_{i}=\frac{c\lambda_{i}}{ab}, \end{array}$$

where  *I*_1_  and  *I*_2_  are the immune strengths of CD4^+^ T cells and macrophages, respectively. Clearly  *R*_0_ > *R*_0_*.

**Lemma**

(i) If  *R*_0_ ≤ 1, then there exists only one uninfected steady state  *E*_0_ = (*x*_1_^0^, 0, *x*_2_^0^, 0,0, 0).

(ii) If  *R*_0_ > 1, then there exist  *E*_0_  and an infected steady state without CTL immune response$\;E_{1}=({\tilde{x}}_{1},{\tilde{y}}_{1},{\tilde{x}}_{2},{\tilde{y}}_{2},\tilde{v},0)$.

(iii) If  *R*_0_* > 1, then there exist  *E*_0_,  *E*_1_, and an infected steady state with CTL immune response  *E*_2_ = (*x*_1_*, *y*_1_*, *x*_2_*, *y*_2_*, v*, z*).

**Proof**

The steady states of (5)–(8) satisfy the following equations:

(19) $$\begin{array}{c} \lambda_{i}-d_{i}x_{i}-\beta_{i}x_{i}v=0, \end{array}$$

(20) $$\begin{array}{c} F_{i}\beta_{i}x_{i}v-ay_{i}-py_{i}z=0, \end{array}$$

(21) $$\begin{array}{c} \sum_{i=1}^{2}kGy_{i}-rv=0, \end{array}$$

(22) $$\begin{array}{c} \sum_{i=1}^{2}cy_{i}z-bz=0. \end{array}$$

From (22) we have

(23) $$\begin{array}{c} \left(\sum_{i=1}^{2}cy_{i}-b\right)z=0. \end{array}$$

Equation (23) has two possible solutions,  *z* = 0  or

(24) $$\begin{array}{c} \sum_{i=1}^{2}cy_{i}-b=0. \end{array}$$

If  *z* = 0, then from (19) and (20) we obtain  *x*_*i*_  and  *y*_*i*_  as

(25) $$\begin{array}{c} x_{i}=\frac{\lambda_{i}}{d_{i}+\beta_{i}v},y_{i}=\frac{F_{i}\beta_{i}\lambda_{i}}{a\left(d_{i}+\beta_{i}v\right)}v, \end{array}$$

and inserting them into (21) we obtain

(26) $$\begin{array}{c} \left(\sum_{i=1}^{2}\frac{kGF_{i}\beta_{i}\lambda_{i}}{a\left(d_{i}+\beta_{i}v\right)}-r\right)v=0. \end{array}$$

Equation (26) has two possible solutions  *v* = 0  or  ∑_*i*=1_^2^(*kGF*_*i*_ *β*_*i*_ *λ*_*i*_/*a*(*d*_*i*_ + *β*_*i*_ *v*)) − *r* = 0.

If  *v* = 0, then substituting it in (25) leads to an uninfected steady state  *E*_0_ = (*x*_1_^0^, 0, *x*_2_^0^, 0,0, 0), where  *x*_*i*_^0^ = *λ*_*i*_/*d*_*i*_,  *i* = 1,2. If  *v* ≠ 0, then we have

(27) $$\begin{array}{c} \sum_{i=1}^{2}\frac{kGF_{i}\beta_{i}\lambda_{i}}{ar\left(d_{i}+\beta_{i}v\right)}-1=0. \end{array}$$

Now we can write (27) as

(28) R011+δ1v+R021+δ2v−1=0,⇒δ1δ2v2+[δ1R01+δ2R02+(1−R0)(δ1+δ2)]v  +(1−R0)=0,

where  *δ*_*i*_ = *β*_*i*_/*d*_*i*_, *i* = 1,2.  The solution of (28) is given by

(29) v±=(−[δ1R01+δ2R02+(1−R0)(δ1+δ2)]  ±([δ1R01+δ2R02+(1−R0)(δ1+δ2)]2  −4δ1δ2(1−R0))1/2)×(2δ1δ2)−1.

Clearly if  *R*_0_ > 1, then  *v* ^+^ > 0  and  *v* ^−^ < 0: then we choose  *v* = *v* ^+^.  Therefore, if  *R*_0_ > 1, then system (5)–(8) has an infected steady state without CTL immune response$\;E_{1}=({\tilde{x}}_{1},{\tilde{y}}_{1},{\tilde{x}}_{2},{\tilde{y}}_{2},\tilde{v},0),$where$\;\tilde{v}=v^{+}\;$and

(30) $$\begin{array}{c} {\tilde{x}}_{i}=\frac{\lambda_{i}}{d_{i}+\beta_{i}\tilde{v}},\;{\tilde{y}}_{i}=\frac{F_{i}\beta_{i}\lambda_{i}}{a\left(d_{i}+\beta_{i}\tilde{v}\right)}\tilde{v},\;i=1,2. \end{array}$$

If  *z* ≠ 0, then from (24) we have

(31) $$\begin{array}{c} \sum_{i=1}^{2}y_{i}^{*}=\frac{b}{c}, \end{array}$$

and inserting it into (21) we obtain

(32) $$\begin{array}{c} v^{*}=\frac{kGb}{rc}. \end{array}$$

From (32) to (19) and (20) we get

(33) $$\begin{array}{c} x_{i}^{*}=\frac{\lambda_{i}rc}{d_{i}rc+\beta_{i}Gkb},\\ y_{i}^{*}=\frac{kGbF_{i}\beta_{i}x_{i}^{*}}{rc\left(a+pz^{*}\right)}=\frac{kGbF_{i}\beta_{i}\lambda_{i}}{\left(a+pz^{*}\right)\left(d_{i}rc+\beta_{i}Gkb\right)}, \end{array}$$

and inserting (31) into (20) we get

(34) $$\begin{array}{c} \Rightarrow z^{*}=\frac{a}{p}\left(\sum_{i=1}^{2}\frac{F_{i}\beta_{i}\lambda_{i}kGc}{a\left(d_{i}rc+\beta_{i}kGb\right)}-1\right)=\frac{a}{p}\left(R_{0}^{*}-1\right). \end{array}$$

We have  *x*_i_* > 0, v* > 0  and if  *R*_0_* > 1, then  z* > 0  and  *y*_i_* > 0. It follows that, if  *R*_0_* > 1, then there exists an infected steady state with CTL immune response  *E*_2_ = (*x*_1_*, *y*_1_*, *x*_2_*, *y*_2_*, v*, z*).

Hence, if  *R*_0_ ≤ 1, then there exists only one steady state  *E*_0_, if  *R*_0_ > 1, then there exist two steady states  *E*_0_  and  *E*_1_, and, if  *R*_0_* > 1, then there exist three steady states  *E*_0_,  *E*_1_, and  *E*_2_.

### 1.4. Global Stability

In this section, we establish the global stability of the three steady states of system (5)–(8) employing the method of Lyapunov functional which is used in [27] for SIR epidemic model with distributed delay. Next, we will use the following notation:  *u* = *u*(*t*)  for any  *u* ∈ {*x*_*i*_, *y*_*i*_, *v*, *z*, *i* = 1,2}. We also define a function  *H* : (0, *∞*)→[0, *∞*)  as  *H*(*u*) = *u* − 1 − ln⁡*u*. It is clear that  *H*(*u*) ≥ 0  for any  *u* > 0  and  *H*  has the global minimum  *H*(1) = 0.

**Theorem**

If  *R*_0_ ≤ 1, then  *E*_0_  is GAS.

**Proof**

Define a Lyapunov functional  *W*_0_  as follows:

(35) W0=∑i=12γi[xi0H(xixi0)+1Fiyi     +βiFi∫0hifi(τ)e−miτ∫−τ0xi(t+θ)                 ×v(t+θ)dθ dτ     +aFiG∫0h3g(τ)e−nτ∫−τ0yi(t+θ)dθ dτ] +v+kGpacz,

where  *γ*_*i*_ = *kGF*_*i*_/*a*,  *i* = 1,2.

The time derivative of  *W*_0_  along the trajectories of (5)–(8) satisfies

(36) dW0dt=∑i=12γi[(1−xi0xi)(λi−dixi−βixiv)     +βiFi∫0hifi(τ)e−miτxi(t−τ)v(t−τ)dτ−aFiyi     −pFiyiz+βiFi∫0hifi(τ)e−miτ              ×(xiv−xi(t−τ)v(t−τ))dτ     +aFiG∫0h3g(τ)e−nτ(yi−yi(t−τ))dτ] +∑i=12k∫0h3g(τ)e−nτyi(t−τ)dτ−rv +∑i=12kGpayiz−kGpbacz.

Collecting terms of (36) we get

(37) dW0dt =∑i=12γi(λi−dixi−λixi0xi+dixi0+βixi0v)   −rv−kGpbacz =∑i=12γiλi(2−xixi0−xi0xi)−kGpbacz−rv   +rv∑i=12kGFiβixi0ar =−∑i=12γidi(xi−xi0)2xi−kGpbacz−rv+rv∑i=12R0i =−∑i=12γidi(xi−xi0)2xi−kGpbacz+(R0−1)rv.

If  *R*_0_ ≤ 1, then  *dW*_0_/*dt* ≤ 0  for all  *x*_1_, *x*_2_, *v*, *z* > 0. By Theorem  5.3.1  in [26], the solutions of system (5)–(8) are limited to  *M*, the largest invariant subset of  {*dW*_0_/*dt* = 0}. Clearly, it follows from (37) that  *dW*_0_/*dt* = 0  if and only if  *x*_*i*_ = *x*_*i*_^0^,  *i* = 1,2,  *v* = 0, and  *z* = 0. Noting that  *M*  is invariant, for each element of  *M*, we have  *v* = 0; then$\;\dot{v}=0$. From (7) we drive that

(38) $$\begin{array}{c} k\int_{0}^{h_{3}}g\left(\tau \right)e^{-n\tau }\sum_{i=1}^{2}y_{i}\left(t-\tau \right)d\tau =0. \end{array}$$

Hence, this yields  ∑_*i*=1_^2^ *y*_*i*_(*t* − *τ*) = 0. Since  *y*_*i*_ ≥ 0  for  *i* = 1,2, then  *y*_1_ = *y*_2_ = 0. Hence  *dW*_0_/*dt* = 0  if and only if  *x*_*i*_ = *x*_*i*_^0^,  *y*_*i*_ = 0,  *i* = 1,2,  *v* = 0, and  *z* = 0. From LaSalle's Invariance Principle,  *E*_0_  is GAS.

**Theorem**

If  *R*_0_ > 1 ≥ *R*_0_*, then  *E*_1_  is GAS.

**Proof**

We construct the following Lyapunov functional:

(39) W1=∑i=12γi[x~iH(xix~i)+1Fiy~iH(yiy~i)     +1Fiβix~iv~     ×∫0hifi(τ)e−miτ∫−τ0H(((xi(t+θ))                   ×(v(t+θ)))                   ×(x~iv~)−1)dθ dτ     +ay~iFiG∫0h3g(τ)e−nτ∫−τ0H(yi(t+θ)y~i)dθ dτ] +v~H(vv~)+kGpacz.

Differentiating with respect to time yields

(40) dW1dt=∑i=12γi[(1−x~ixi)(λi−dixi−βixiv)+1Fi(1−y~iyi)     ×(βi∫0hifi(τ)e−miτxi(t−τ)v(t−τ)dτ        −ayi−pyiz)     +βiFi∫0hifi(τ)e−miτ        ×(xiv−xi(t−τ)v(t−τ)           +x~iv~ln⁡(xi(t−τ)v(t−τ)xiv))dτ     +aFiG∫0h3g(τ)e−nτ(yi−yi(t−τ)                  +y~iln⁡(yi(t−τ)yi))dτ]    +(1−v~v)(∑i=12k∫0h3g(τ)e−nτyi(t−τ)dτ−rv)  +kGpac(∑i=12cyiz−bz).

Collecting terms we obtain

(41) dW1dt=∑i=12γi[λi−dixi−λix~ixi+dix~i+βix~iv     −βiy~iFiyi∫0hifi(τ)e−miτxi(t−τ)v(t−τ)dτ     +aFiy~i+pFiy~iz     +1Fiβix~iv~∫0hifi(τ)e−miτ            ×ln⁡(xi(t−τ)v(t−τ)xiv)dτ     +ay~iFiG∫0h3g(τ)e−nτln⁡(yi(t−τ)yi)dτ]  −rv−v~v∑i=12k∫0h3g(τ)e−nτyi(t−τ)dτ  +rv~−kGpbacz.

From (19)–(21) we have

(42) $$\begin{array}{c} \lambda_{i}=d_{i}{\tilde{x}}_{i}+\beta_{i}{\tilde{x}}_{i}\tilde{v},\;\;F_{i}\beta_{i}{\tilde{x}}_{i}\tilde{v}=a{\tilde{y}}_{i},\;\;r\tilde{v}=\sum_{i=1}^{2}Gk{\tilde{y}}_{i}. \end{array}$$

Using (42) and the following equality:

(43) $$\begin{array}{c} rv=r\tilde{v}\frac{v}{\tilde{v}}=\frac{v}{\tilde{v}}\sum_{i=1}^{2}Gk{\tilde{y}}_{i}=\frac{v}{\tilde{v}}\sum_{i=1}^{2}\frac{\gamma_{i}a}{F_{i}}{\tilde{y}}_{i}=\frac{v}{\tilde{v}}\sum_{i=1}^{2}\gamma_{i}\beta_{i}{\tilde{x}}_{i}\tilde{v}, \end{array}$$

we obtain

(44) dW1dt=∑i=12γi[dix~i+βix~iv~−dixi−x~ixi(dix~i+βix~iv~)     +dix~i+βix~iv~vv~     −1Fiβix~iv~∫0hifi(τ)e−miτy~ixi(t−τ)v(t−τ)yix~iv~dτ     +βix~iv~+pFiy~iz     +1Fiβix~iv~∫0hifi(τ)e−miτ            ×ln⁡(xi(t−τ)v(t−τ)xiv)dτ     +1Gβix~iv~i∫0h3g(τ)e−nτln⁡(yi(t−τ)yi)dτ     −βix~iv~vv~−1Gβix~iv~∫0h3g(τ)e−nτv~yi(t−τ)vy~idτ     +βix~iv~]−kGpbacz.

Then collecting terms of (44) and using the following equalities:

(45) ln⁡(xi(t−τ)v(t−τ)xiv)=ln⁡(y~ixi(t−τ)v(t−τ)yix~iv~) +ln⁡(x~ixi)+ln⁡(v~yivy~i), i=1,2,ln⁡(yi(t−τ)yi)=ln⁡(vy~iv~yi)+ln⁡(v~yi(t−τ)vy~i), i=1,2,

we obtain

(46) dW1dt=∑i=12γi[dix~i(2−x~ixi−xix~i)+βix~iv~(1−x~ixi)+2βix~iv~     −1Fiβix~iv~∫0hifi(τ)e−miτ            ×y~ixi(t−τ)v(t−τ)yix~iv~dτ     +1Fiβix~iv~∫0hifi(τ)e−miτ            ×(ln⁡(y~ixi(t−τ)v(t−τ)yix~iv~)               +ln⁡(x~ixi)+ln⁡(v~yivy~i))dτ     +1Gβix~iv~∫0h3g(τ)e−nτ            ×(ln⁡(vy~iv~yi)               +ln⁡(v~yi(t−τ)vy~i))dτ     −1Gβix~iv~∫0h3g(τ)e−nτv~yi(t−τ)vy~idτ]    +∑i=12kGpay~iz−kGpbacz.

Equation (46) can be rewritten as

(47) dW1dt=∑i=12γi[dix~i(2−x~ixi−xix~i)−βix~iv~(x~ixi−1−ln⁡(x~ixi))     −1Fiβix~iv~∫0hifi(τ)e−miτ            ×(y~ixi(t−τ)v(t−τ)yix~iv~−1               −ln⁡(y~ixi(t−τ)v(t−τ)yix~iv~))dτ     −1Gβix~iv~∫0h3g(τ)e−nτ(v~yi(t−τ)vy~i−1                   −ln⁡(v~yi(t−τ)vy~i))dτ]    +kGpa(∑i=12y~i−bc)z.

We can rewrite  *dW*_1_/*dt*  as:

(48) dW1dt=−∑i=12γi[di(xi−x~i)2xi+βix~iv~H(x~ixi)      +1Fiβix~iv~      ×∫0hifi(τ)e−miτH(((y~ixi(t−τ))                 ×(v(t−τ)))                 ×(yix~iv~)−1)dτ      +1Gβix~iv~∫0h3g(τ)e−nτH(v~yi(t−τ)vy~i)dτ]     +kGpa(∑i=12y~i−bc)z.

Now we show that, if  *R*_0_* ≤ 1, then$\;\sum_{i=1}^{2}{\tilde{y}}_{i}<b/c$. Assume that  *R*_0_* ≤ 1; then

(49) $$\begin{array}{c} \sum_{i=1}^{2}\frac{kGcF_{i}\beta_{i}\lambda_{i}}{arcd_{i}+a\beta_{i}Gkb}\leq 1. \end{array}$$

From (27) we have

(50) $$\begin{array}{c} \sum_{i=1}^{2}\frac{kGF_{i}\beta_{i}\lambda_{i}}{ard_{i}+a\beta_{i}r\tilde{v}}=1,\\ \Rightarrow \sum_{i=1}^{2}\frac{kGcF_{i}\beta_{i}\lambda_{i}}{arcd_{i}+a\beta_{i}cr\tilde{v}}=1. \end{array}$$

Comparing (49) and (50) we get

(51) $$\begin{array}{c} \sum_{i=1}^{2}\frac{kGcF_{i}\beta_{i}\lambda_{i}}{arcd_{i}+a\beta_{i}Gkb}\leq \sum_{i=1}^{2}\frac{kGcF_{i}\beta_{i}\lambda_{i}}{arcd_{i}+a\beta_{i}cr\tilde{v}}. \end{array}$$

Then

(52) $$\begin{array}{c} \tilde{v}\leq \frac{Gkb}{rc}. \end{array}$$

Using (42) we have

(53) $$\begin{array}{c} \frac{Gk}{r}\sum_{i=1}^{2}\tilde{y_{i}}=\tilde{v}\leq \frac{Gkb}{rc}. \end{array}$$

Then

(54) $$\begin{array}{c} \sum_{i=1}^{2}\tilde{y_{i}}\leq \frac{b}{c}. \end{array}$$

Now if  *R*_0_ > 1, then$\;{\tilde{x}}_{i},{\tilde{y}}_{i},\tilde{v}>0.\;$It follows that, if  *R*_0_ > 1 ≥ *R*_0_*, then  *dW*_1_/*dt* ≤ 0. By Theorem  5.3.1  in [26], the solutions of system (5)–(8) are limited to  *M*, the largest invariant subset of  {*dW*_1_/*dt* = 0}. It can be seen that  *dW*_1_/*dt* = 0  if and only if$\;x_{i}={\tilde{x}}_{i}$,  *z* = 0, and  *H* = 0: that is,

(55) y~ixi(t−τ)v(t−τ)yix~iv~=v~yi(t−τ)vy~i=1       for almost all τ∈[0,h].

If$\;x_{i}={\tilde{x}}_{i}$, then from (19) we have$\;v=\tilde{v}\;$and from (55) we have$\;y_{i}={\tilde{y}}_{i}$. It follows that  *dW*_1_/*dt* = 0  at  *E*_1_. LaSalle's Invariance Principle implies the global stability of  *E*_1_.

**Theorem**

If  *R*_0_ > *R*_0_* > 1, then  *E*_2_  is GAS.

**Proof**

We construct the following Lyapunov functional:

(56) W2=∑i=12γi[xi∗H(xixi∗)+1Fiyi∗H(yiyi∗)     +1Fiβixi∗v∗     ×∫0hifi(τ)e−miτ∫−τ0H(((xi(t+θ))                ×(v(t+θ)))                ×(xi∗v∗)−1)dθ dτ     +(ayi∗+pyi∗z∗)FiG∫0h3g(τ)e−nτ                ×∫−τ0H(yi(t+θ)yi∗)dθ dτ]   +(1+paz∗)v∗H(vv∗)+kGpacz∗H(zz∗).

Differentiating with respect to time yields

(57) dW2dt=∑i=12γi[(1−xi∗xi)(λi−dixi−βixiv)     +1Fi(1−yi∗yi)(βi∫0hifi(τ)e−miτxi(t−τ)                ×v(t−τ)dτ−ayi−pyiz)     +βiFi∫0hifi(τ)e−miτ         ×(xiv−xi(t−τ)v(t−τ)            +xi∗v∗ln⁡(xi(t−τ)v(t−τ)xiv))dτ     +aFiG∫0h3g(τ)e−nτ          ×(yi−yi(t−τ)+yi∗ln⁡(yi(t−τ)yi))dτ     +pFiG∫0h3g(τ)e−nτ          ×(yiz∗−yi(t−τ)z∗            +yi∗z∗ln⁡(yi(t−τ)yi))dτ]  +(1+paz∗)(1−v∗v)  ×(∑i=12k∫0h3g(τ)e−nτyi(t−τ)dτ−rv)  +kGpac(1−z∗z)(∑i=12cyiz−bz).

Collecting terms we obtain

(58) dW2dt=∑i=12γi[λi−dixi−λixi∗xi+dixi∗+βixi∗v     −βiyi∗Fiyi∫0hifi(τ)e−miτxi(t−τ)v(t−τ)dτ     +aFiyi∗+pFiyi∗z+1Fiβixi∗v∗     ×∫0hifi(τ)e−miτln⁡(xi(t−τ)v(t−τ)xiv)dτ     +ayi∗FiG∫0h3g(τ)e−nτln⁡(yi(t−τ)yi)dτ     +pyi∗z∗FiG∫0h3g(τ)e−nτln⁡(yi(t−τ)yi)dτ]−rv  −v∗v∑i=12k∫0h3g(τ)e−nτyi(t−τ)dτ+rv∗−pz∗arv  −v∗v∑i=12ka∫0h3g(τ)e−nτpyi(t−τ)z∗dτ+pz∗arv∗  −kGpbacz+kGpbacz∗.

Using (19)–(21), we have equalities

(59) $$\begin{array}{c} rv=rv^{*}\left(\frac{v}{v^{*}}\right)=\frac{v}{v^{*}}\sum_{i=1}^{2}Gky_{i}^{*}=\frac{v}{v^{*}}\sum_{i=1}^{2}\frac{\gamma_{i}}{F_{i}}ay_{i}^{*},\\ \frac{pz^{*}}{a}rv^{*}=\sum_{i=1}^{2}\frac{kG}{a}py_{i}^{*}z^{*}=\sum_{i=1}^{2}\frac{\gamma_{i}}{F_{i}}py_{i}^{*}z^{*},\\ \frac{pz^{*}}{a}rv=\frac{pz^{*}}{a}rv^{*}\left(\frac{v}{v^{*}}\right)=\frac{v}{v^{*}}\sum_{i=1}^{2}\frac{\gamma_{i}}{F_{i}}py_{i}^{*}z^{*},\\ \frac{kGpb}{ac}z^{*}=\sum_{i=1}^{2}\frac{kG}{a}py_{i}^{*}z^{*}=\sum_{i=1}^{2}\frac{\gamma_{i}}{F_{i}}py_{i}^{*}z^{*},\;\text{since}\;\;\sum_{i=1}^{2}y_{i}^{*}=\frac{b}{c},\\ \frac{kGpb}{ac}z=\frac{kGpb}{ac}z^{*}\left(\frac{z}{z^{*}}\right)=\sum_{i=1}^{2}\frac{\gamma_{i}}{F_{i}}py_{i}^{*}z. \end{array}$$

We obtain

(60) dW2dt=∑i=12γi[dixi∗+βixi∗v∗−dixi−xi∗xi(dixi∗+βixi∗v∗)     +dixi∗+βixi∗v∗vv∗−1Fiβixi∗v∗     ×∫0hifi(τ)e−miτyi∗xi(t−τ)v(t−τ)yixi∗v∗dτ     +ayi∗Fi+pyi∗zFi     +1Fiβixi∗v∗∫0hifi(τ)e−miτ             ×ln⁡(xi(t−τ)v(t−τ)xiv)dτ     +ayi∗FiG∫0h3g(τ)e−nτln⁡(yi(t−τ)yi)dτ     +pyi∗z∗FiG∫0h3g(τ)e−nτln⁡(yi(t−τ)yi)dτ     −ayi∗Fivv∗−ayi∗FiG∫0h3g(τ)e−nτ                ×yi(t−τ)v∗yi∗vdτ     +ayi∗Fi−pyi∗z∗Fivv∗     −pyi∗z∗FiG∫0h3g(τ)e−nτyi(t−τ)v∗yi∗vdτ     +pyi∗z∗Fi−pyi∗zFi+pyi∗z∗Fi].

Then collecting terms of (60)

(61) dW2dt=∑i=12γi[dixi∗(2−xi∗xi−xixi∗)+βixi∗v∗(1−xi∗xi)    +βixi∗v∗vv∗    −1Fiβixi∗v∗∫0hifi(τ)e−miτ            ×yi∗xi(t−τ)v(t−τ)yixi∗v∗dτ    +2Fi(ayi∗+pyi∗z∗)    +1Fiβixi∗v∗∫0hifi(τ)e−miτ            ×ln⁡(xi(t−τ)v(t−τ)xiv)dτ    +1FiG(ayi∗+pyi∗z∗)∫0h3g(τ)e−nτ                 ×ln⁡(yi(t−τ)yi)dτ    −1FiG(ayi∗+pyi∗z∗)∫0h3g(τ)e−nτ                ×yi(t−τ)v∗yi∗vdτ    −1Fi(ayi∗+pyi∗z∗)vv∗].

Using the following equalities:

(62) βixi∗v∗=1Fi(ayi∗+pyi∗z∗),ln⁡(xi(t−τ)v(t−τ)xiv)=ln⁡(yi∗xi(t−τ)v(t−τ)yixi∗v∗) +ln⁡(xi∗xi)+ln⁡(v∗yivyi∗), i=1,2,ln⁡(yi(t−τ)yi)=ln⁡(vyi∗v∗yi)+ln⁡(v∗yi(t−τ)vyi∗), i=1,2,

we obtain

(63) dW2dt=∑i=12γi[dixi∗(2−xi∗xi−xixi∗)+βixi∗v∗     ×(1−xi∗xi)+2βixi∗v∗     −1Fiβixi∗v∗∫0hifi(τ)e−miτ             ×yi∗xi(t−τ)v(t−τ)yixi∗v∗dτ     +1Fiβixi∗v∗∫0hifi(τ)e−miτ             ×(ln⁡(yi∗xi(t−τ)v(t−τ)yixi∗v∗)               +ln⁡(xi∗xi)+ln⁡(v∗yivyi∗))dτ     +1Gβixi∗v∗∫0h3g(τ)e−nτ             ×(ln⁡(vyi∗v∗yi)                +ln⁡(v∗yi(t−τ)vyi∗))dτ     −1Gβixi∗v∗∫0h3g(τ)e−nτv∗yi(t−τ)vyi∗dτ].

Equation (63) can be rewritten as

(64) dW2dt=∑i=12γi[dixi∗(2−xi∗xi−xixi∗)     −βixi∗v∗(xi∗xi−1−ln⁡(xi∗xi))     −1Fiβixi∗v∗∫0hifi(τ)e−miτ             ×(yi∗xi(t−τ)v(t−τ)yixi∗v∗−1              −ln⁡(yi∗xi(t−τ)v(t−τ)yixi∗v∗))dτ     −1Gβixi∗v∗∫0h3g(τ)e−nτ             ×(v∗yi(t−τ)vyi∗−1               −ln⁡(v∗yi(t−τ)vyi∗))dτ]=−∑i=12γi[di(xi−xi∗)2xi+βixi∗v∗H(xi∗xi)      +1Fiβixi∗v∗∫0hifi(τ)e−miτ              ×H(yi∗xi(t−τ)v(t−τ)yixi∗v∗)dτ      +1Gβixi∗v∗      ×∫0h3g(τ)e−nτH(v∗yi(t−τ)vyi∗)dτ].

It can be easily seen that, if  *x*_i_*, *y*_i_*, v* > 0,  *i* = 1,2, then  *dW*_2_/*dt* ≤ 0. By Theorem  5.3.1  in [26], the solutions of system (5)–(8) are limited to  *M*, the largest invariant subset of  {*dW*_2_/*dt* = 0}. It can be seen that  *dW*_2_/*dt* = 0  if and only if  *x*_*i*_ = *x*_i_*  and  *H* = 0; that is,

(65) yi∗xi(t−τ)v(t−τ)yixi∗v∗=v∗yi(t−τ)vyi∗=1       for almost all τ∈[0,h].

If  *x*_*i*_ = *x*_i_*, then from (19) we have  *v* = v*  and from (65) we have  *y* = y*.  Moreover, from (20) we have

(66) $$\begin{array}{c} 0=\beta_{i}F_{i}x_{i}^{*}v^{*}-ay_{i}^{*}-py_{i}^{*}z\;\;\Rightarrow z=\frac{\beta_{i}F_{i}x_{i}^{*}v^{*}}{py_{i}^{*}}-\frac{a}{p}=z^{*}. \end{array}$$

Hence  *dW*_2_/*dt* = 0  at  *E*_2_. LaSalle's Invariance Principle implies the global stability of  *E*_2_.

## 2. Conclusion

In this paper, we have proposed an HIV infection model describing the interaction of the HIV with two classes of target cells, CD4^+^ T cells and macrophages, taking into account the CTL immune response. Two types of distributed time delays have been incorporated into the model to describe the time needed for infection of target cell and virus replication. The global stability of the three steady states of the model has been established by constructing suitable Lyapunov functionals and using LaSalle's Invariant Principle. We have proven that

- if  *R*_0_ ≤ 1, then the uninfected steady state  *E*_0_  is GAS;
- if  *R*_0_ > 1 ≥ *R*_0_*, then the infected steady state without CTL immune response  *E*_1_  is GAS;
- if  *R*_0_ > *R*_0_* > 1, then the infected steady state with CTL immune response  *E*_2_  is GAS.
